# Exploring the potential link between ADHD, anxiety, autism, and cancer risk

**DOI:** 10.3389/fonc.2025.1688883

**Published:** 2025-12-15

**Authors:** Shreya Wajapeyee, Kristina Ford, Romi Gupta

**Affiliations:** 1 Department of Pediatrics Division of Developmental-Behavioral Pediatrics, University of Alabama at Birmingham (UAB), Birmingham, AL, United States; 2 Department of Biochemistry and Molecular Genetics, University of Alabama at Birmingham (UAB), Birmingham, AL, United States; 3 O’Neal Comprehensive Cancer Center, University of Alabama at Birmingham (UAB), Birmingham, AL, United States

**Keywords:** AD, ADHD, anxiety, ASD, autism, cancer

## Abstract

Neurodevelopmental and mental health disorders such as autism spectrum disorder (ASD), attention-deficit/hyperactivity disorder (ADHD), and anxiety disorders (AD) are increasingly being studied for their potential links to cancer risk. In this review, we present evidence indicating that ASD alone is not strongly associated with an increased risk of cancer. However, elevated risk is observed in subgroups with co-occurring intellectual disability or congenital anomalies. For ADHD, no direct biological link to cancer has been established, though behavioral factors and medication use remain areas of concern. Anxiety disorders may contribute indirectly to cancer risk through chronic stress and associated health behaviors, with some studies suggesting increased risk for specific cancers, such as prostate or urological cancers. Overall, this review highlights that increased cancer risk appears to be more closely related to co-occurring conditions and lifestyle factors than to the primary diagnoses themselves.

## Introduction

Neurodevelopmental and mental health disorders such as autism spectrum disorder (ASD), attention-deficit/hyperactivity disorder (ADHD), and anxiety disorders (AD) affect millions of individuals worldwide and have profound impacts on cognitive, emotional, and social functioning (1, 2). While these conditions are primarily associated with learning, behavior, and mental health issues, there is increasing evidence that they may also influence long-term physical health outcomes, particularly cancer risk (3). The complex interplay between genetic, biological, behavioral, and environmental factors in these disorders raises important questions about whether individuals with these diagnoses face an elevated risk of developing cancer. In this review, we explore whether these conditions are associated with an increased risk for cancer.

## Autism spectrum disorder and cancer risk

The relationship between autism spectrum disorder and cancer risk has attracted increasing attention due to overlapping molecular and genetic factors implicated in both neurodevelopmental disorders and tumorigenesis (4–7). Nussinov et al. demonstrated that neurodevelopmental disorders and cancer involve alterations in key signaling pathways that regulate cell growth, differentiation, chromatin structure, and synaptic development. Important overlaps include chromatin-remodeling genes such as ARID1B, SMARCA4, and SMARCB1, which are part of the SWI/SNF complex and function as tumor suppressors but also contribute to ASD and intellectual disability when mutated. Major signaling pathways, particularly the PI3K-AKT-mTOR signaling pathway, are affected through mutations in PTEN, TSC1, and TSC2 gene contributing to abnormal neural development and increased cancer predisposition. MAPK/ERK signaling, including PAK1-regulated pathway, also has dual role, including influencing neuronal migration and synaptic plasticity in ASD while promoting proliferation and invasion in cancer. Additional genes such as CHD8, ATM, ATRX, SETD2, and DNMT3A highlight shared mechanisms involving chromatin regulation, DNA repair, and cell-cycle control.

Similarly, Crawley et al. reported substantial genetic overlap between ASD and cancer, identifying more than 40 shared genes involved in chromatin remodeling, transcriptional regulation, synaptic development, and cell-growth signaling pathways. Overlapping pathways such as PI3K-AKT-mTOR, MAPK/ERK, WNT, and NOTCH may influence either neurodevelopment or tumorigenesis depending on the cellular context. Important genes with dual roles such as PTEN, TSC1, TSC2, CHD8, ARID1B, NF1, and HRAS were also identified.

Gabrielli et al. further expanded this connection by identifying 138 genes shared between ASD and cancer. Many of these genes participate in major signaling pathways such as ERK/MAPK, PI3K-AKT-mTOR, GPCR signaling, and growth-factor signaling (HGF, TGF-β) pathways. Overlapping genes included chromatin-remodeling regulators (CHD8, ARID1B, ATRX), tumor-suppressor genes (PTEN, NF1, TSC1, TSC2), and transcription factors (ADNP, FOXP1, TCF7L2). Functional enrichment analysis revealed common alterations in transcriptional regulation, chromatin organization, kinase activity, and β-catenin binding, supporting the idea that alterations in these pathways contributes to both ASD and cancer.

Fores-Martos et al. compared gene-expression profiles of ASD frontal cortex tissues with 22 cancer types. They found that brain, thyroid, kidney, and pancreatic cancers share gene-expression changes in the same direction as ASD, while lung and prostate cancers show opposite deregulation patterns. Common biological processes included immune dysfunction, impaired oxidative phosphorylation and ATP synthesis, and altered signaling pathways. Notably, brain and kidney cancers showed PI3K/AKT/mTOR dysregulation similar to that observed in ASD. These findings suggest that certain cancers may share direct molecular links with ASD, while others may show inverse associations. Overall, growing molecular and transcriptomic evidence indicates substantial shred genetic mechanisms between ASD and cancer, raising the possibility that some cancer-targeted therapies may have relevance for specific ASD subtypes treatments.

Studies examining epidemiological associations have shown that while individuals with ASD generally do not exhibit a significantly increased cancer risk, specific subgroups especially those with rare genetic mutations or co-occurring conditions may be more vulnerable (3). A large population-based cohort study in the Nordic countries analyzed 2.3 million individuals born in Sweden between 1987 and 2013, with follow-up through 2016, to assess early-life cancer risk in relation to ASD. ASD diagnoses were obtained from the Swedish National Patient Register, and cancer risk was measured using logistic regression adjusted for multiple confounders. Sibling comparisons were used to control for familial confounding and genome-wide association-based genetic correlation analyses to examine potential polygenic pleiotropy.

Overall, individuals with ASD showed a modestly increased risk of any cancer compared to those without ASD (odds ratio [OR] = 1.3; 95% confidence interval [CI]: 1.2–1.5). This increased risk was most pronounced in individuals with narrowly defined autistic disorder (OR = 1.7; 95% CI: 1.3–2.1) and in those with ASD plus comorbid birth defects (OR = 2.1; 95% CI: 1.5–2.9) or both birth defects and intellectual disability (ID) (OR = 4.8; 95% CI: 3.4–6.6). A nonsignificant trend was observed for ASD with ID alone (OR = 1.4; 95% CI: 0.9–2.1). Importantly, ASD without comorbid birth defects or ID was not associated with increased cancer risk (OR = 1.0; 95% CI: 0.8–1.2). Sibling and genetic correlation analyses showed minimal evidence of familial or polygenic confounding, suggesting the observed increase in cancer risk is attributable primarily to co-occurring birth defects and/or intellectual disability rather than ASD itself. Thus, this study showed that ASD alone does not confer a higher early-life cancer risk; rather, cancer risk elevation is linked to associated conditions such as birth defects and intellectual disability (3).

Similarly, a Taiwanese nationwide cohort study was performed to investigate whether individuals with autism have an increased risk for cancer compared with the general population. In 8,438 autistic patients, 20 were diagnosed with cancer, corresponding to a standardized incidence ratio (SIR) of 1.94 for overall cancer risk in individuals with ASD. The highest risk was observed among adolescents aged 15–19 (SIR = 3.58) and significantly elevated risks was noticed for genitourinary cancers (SIR 4.15), including ovarian cancer (SIR = 9.21) Cancer occurrence was higher than expected in males (SIR 1.95), but the increase in females (SIR 1.91) was not statistically significant (8).

## ADHD and cancer risk

The relationship between Attention-Deficit/Hyperactivity Disorder (ADHD) and cancer risk is also under investigation. Current evidence does not support a direct biological association between ADHD and an elevated risk of cancer (9). ADHD is a common neurodevelopmental disorder characterized by patterns of lack of attention, impulsivity, and hyperactivity, often persisting into adulthood (10). While ADHD itself is not classified as a cancer predisposition syndrome, ongoing studies are investigating whether underlying neurodevelopmental factors, associated behaviors, or treatment regimens might contribute to an increased cancer risk.

A significant area of focus has been the potential long-term impact of medications, such as methylphenidate and amphetamines, which are commonly prescribed to manage ADHD symptoms. A large, representative 17-year cohort study found no association between long-term methylphenidate use in children and increased cancer risk (11). Conversely, another study examined the potential cytogenetic effects of methylphenidate (Ritalin) in children treated for ADHD. In 12 pediatric patients, significant increases in chromosome aberrations, sister chromatid exchanges, and micronuclei were observed after three months of treatment. These findings suggest methylphenidate may cause genetic damage, raising concerns about potential long-term health risks, including increased cancer susceptibility, and highlight the need for further investigation (12).

Several studies have also examined indirect cancer risks and the behavioral and lifestyle factors commonly observed in individuals with ADHD. Individuals with ADHD have high-risk health behaviors such as tobacco use, substance abuse, poor diet, and physical inactivity, all well-established contributors to cancers including lung, liver, and colorectal cancers. For example, a retrospective cohort study conducted in Taiwan analyzed 798 individuals with ADHD and 2,394 matched controls (2000-2013) to assess colorectal cancer (CRC) risk. CRC incidence was evaluated using Kaplan–Meier analysis, and Cox proportional hazards models were applied to estimate hazard ratios (HRs). Findings suggested an increased risk of CRC among individuals with ADHD, although further study is necessary to confirm this association and elucidate the underlying mechanisms (13).

Another study used Mendelian randomization (MR) to evaluate potential causal relationships between various psychiatric disorders such as schizophrenia, bipolar disorder (BD), major depressive disorder (MDD), Attention-Deficit/Hyperactivity Disorder (ADHD), autism spectrum disorder (ASD), and anxiety disorder (AD) and breast cancer (BC). Mendelian Randomization (MR) is a statistical genetic method that uses naturally occurring genetic variants to determine whether an observed association between a risk factor (such as a psychiatric disorder) and an outcome (such as breast cancer) is causal rather than due to confounding or reverse causation. MR is especially useful in this study because psychiatric disorders and cancer share many confounders including lifestyle factors, medication use, stress, and socioeconomic status, which make observational findings difficult to interpret. By using genetic variants as natural experiments, MR reduces these biases and helps determine whether psychiatric disorders genuinely contribute to breast cancer risk.

In this study, the authors used genome-wide significant single nucleotide polymorphisms (SNPs) from large Genome-Wide Association Study (GWAS) datasets as genetic variants for schizophrenia, bipolar disorder, major depressive disorder, Attention-Deficit/Hyperactivity Disorder, autism spectrum disorder, and anxiety disorder. These SNPs reflect inherited genetic predisposition to each disorder. When tested against breast cancer GWAS data, the MR revealed no causal link between schizophrenia, BD, MDD, ADHD, or ASD and breast cancer. However, SNPs associated with anxiety disorder showed a positive causal relationship between anxiety disorder and breast cancer (14), suggesting that biological pathways related to anxiety such as stress-response, neurotransmitter regulation, or neuroendocrine mechanisms may influence cancer risk. It is important to note that despite its strengths, MR has limitations. Genetic variants used for study must affect the outcome only in a specific condition, otherwise, pleiotropy can bias results. Another limitation include that MR reflects lifetime genetic predisposition rather than environmentally triggered risk, and weak genetic variability can reduce accuracy.

Similarly, a different study used genome-wide association data and two-sample MR to investigate genetic links between psychiatric disorders and lung cancer. Significant positive genetic correlations were found between most psychiatric disorders and both smoking and lung cancer, except for autism. MR analyses showed that genetic liability to schizophrenia, depression, ADHD, and insomnia increases lung cancer risk. Specifically, ADHD was associated with higher risks of squamous cell carcinoma and small cell lung cancer, even after adjusting for smoking. The study suggested that managing psychiatric disorders, especially ADHD, may help lower lung cancer risk, though results are limited to individuals of European ancestry (15).

In conclusion, while ADHD does not appear to increase cancer risk through direct biological or genetic pathways, indirect factors particularly modifiable health behaviors and disparities in healthcare access may contribute to a modest increase in cancer incidence in this population. Continued long-term cohort studies and deeper exploration of biological and behavioral mechanisms are essential to clarify the potential link between ADHD and cancer risk.

## Anxiety disorders and cancer risk

Studies examining the relationship between anxiety disorders and cancer risk have produced mixed results. Anxiety disorders including generalized anxiety disorder (GAD), panic disorder, and social anxiety disorder are among the most common psychiatric conditions worldwide. They are characterized by excessive worry, hyperarousal, and often chronic stress (16). One proposed mechanism linking anxiety to cancer involves chronic activation of the stress response, particularly via the hypothalamic-pituitary-adrenal (HPA) axis. This activation can lead to sustained elevations in cortisol, immune dysregulation, oxidative stress, and low-grade systemic inflammation, all of which have been implicated in tumor development and progression (17). Persistent anxiety has also been associated with reduced natural killer cell activity and cytokine dysfunction (18), and chronic stress exposure may contribute to tumor initiation in certain cancers (19). Further in-depth study is needed to clarify how stress hormones might trigger tumorigenic pathways in susceptible individuals.

A study using data from Taiwan’s National Health Insurance (NHI) system examined the association between anxiety disorder (AD) and cancer risk. The cohort included 24,066 patients diagnosed with AD, each frequency-matched by age and sex to four individuals from the general population without AD. Cox proportional hazards regression analysis was used to evaluate the effect of AD on cancer development. Overall, the study found that patients with AD had only a 1% higher risk of developing cancer compared to controls, a difference that was not statistically significant (hazard ratio [HR] = 1.01; 95% confidence interval [CI]: 0.95–1.07). However, when analyzing specific cancer types, the results showed a significantly increased risk of prostate cancer in male patients with AD (HR = 1.32; 95% CI: 1.02–1.71). In contrast, female patients with AD had a marginally reduced risk of developing cervical cancer (HR = 0.72; 95% CI: 0.51–1.03). A key limitation of the study was the lack of information on lifestyle and behavioral factors in the NHI database, such as smoking, alcohol consumption, and other relevant health behaviors, which may confound the results. Despite not finding a significant association between AD and overall cancer risk, the findings suggest that AD may be linked to an increased prostate cancer risk and a potentially decreased risk of cervical cancer among Taiwanese patients (20).

Another study, using data from the Taiwan Longitudinal Health Insurance Database 2005, investigated the 5-year risk of urological cancers in patients with anxiety disorders (ADs) compared to matched controls without ADs. Patients were matched one-to-one by sex, age, and year of recruitment. Urological cancers occurred in 0.54% of AD patients versus 0.13% of controls. After adjusting for sociodemographic factors, comorbidities, and substance use disorders, patients with ADs had a significantly increased risk of developing urological cancers (adjusted HR = 3.67; 95% CI: 2.85–4.72). The risk remained elevated in both males (HR = 3.82) and females (HR = 3.47) with ADs. The study concluded that anxiety disorders are associated with a more than threefold increased risk of urological cancers over five-year period (21).

In addition to biological mechanisms, behavioral factors also play a significant role. Chronic anxiety is often linked to increased tobacco use, alcohol misuse, poor diet, sedentary lifestyle, and lower adherence to preventive healthcare, all of which are known contributors to elevated cancer risk. Moreover, anxiety can lead to delays in cancer diagnosis and treatment, particularly in early-stage disease (22).

In summary, while anxiety disorders do not directly cause cancer, chronic anxiety may increase cancer risk through a combination of biological stress mechanisms and unhealthy behaviors. Close monitoring, promotion of preventive care, and further in-depth studies are essential to better understand and manage these potential risks.

## Shared mechanisms and overlapping risk factors

While autism, ADHD, and anxiety are distinct conditions, they may share overlapping biological pathways that intersect with cancer biology (7, 23) (4). These conditions also frequently co-occur, making it challenging to isolate their individual contributions to health risks. For example, individuals with autism often experience high levels of anxiety, and those with ADHD frequently present with comorbid mood or anxiety disorders (24). This comorbidity underscores the need for holistic models to better understand the molecular mechanisms that may predispose these populations to an increased risk of cancer and to inform more effective, integrated care strategies.

## Conclusion

Current evidence suggests that autism spectrum disorder alone is not associated with a significantly increased risk of cancer. However, individuals with ASD who also have co-occurring intellectual disability, congenital anomalies, or rare genetic mutations may exhibit elevated cancer susceptibility. Similar trends are observed in ADHD and anxiety disorders, where indirect factors such as lifestyle behaviors, chronic stress, and healthcare disparities may contribute modestly to cancer risk. These findings as summarized in **Table 1** highlight the importance of examining comorbidities and shared biological pathways rather than attributing cancer risk to the primary neurodevelopmental or psychiatric diagnosis alone.

**Table 1:** Summary table outlining each disorder and its potential association with cancer risk.

Neurodegenerative disorder	Cancer risk association	Contributing factors
Autism spectrum disorder (ASD)	Modest increase only in subgroups (birth defect, intellectual disability), none in ASD alone	Genetic variant, birth defects, intellectual disability (ID), sex differences
Attention deficit hyperactivity disorder (ADHD)	No direct links, possible indirect risk	Smoking, substance abuse, poor diet, healthcare issues
Anxiety disorder (AD)	No direct links have been identified, but possible indirect risks exist. One study reported an increased risk for prostate cancer and urological cancers.	Chronic stress, hypothalamic-pituitary-adrenal (HPA) activation, immune dysfunction

## Future direction

Future research should aim to identify high-risk subgroups by using stratified analyses that consider genetic, clinical, and behavioral differences. Longitudinal cohort studies following individuals across their lifespan are needed to better understand late-onset cancer risk. Integrative multi-omic approaches may reveal shared molecular mechanisms between neurodevelopmental disorders and cancer. Focused strategies to address modifiable risk factors such as tobacco use, lack of exercise, and unhealthy eating habits are crucial for effective prevention. These efforts should be paired with improved healthcare access and early cancer screening to reduce cancer burden in vulnerable populations.

The possible link between neurodevelopmental conditions and cancer risk underscores the need for more integrated healthcare strategies. Preventive care, including routine cancer screenings, may be underutilized in these groups. Patients with high-risk genetic syndromes, such as PTEN mutations associated with autism, could benefit from personalized surveillance and genetic counseling.

## Methodology

### Bioinformatics analysis for identifying appropriate research articles-

We searched PubMed (MEDLINE) for studies examining neurological disorders and their association with cancer risk. The search strategy combined MeSH terms and free-text keywords related to autism spectrum disorder (ASD), attention-deficit/hyperactivity disorder (ADHD), anxiety disorder (AD), and cancer risk, and included publications from year 2000 to 2025. Original research articles, book excerpt and review papers were included, while editorials and conference abstracts were excluded. In addition, the reference lists of key articles were manually reviewed to identify any relevant studies not captured in the initial search.

## Glossary

AD: Anxiety Disorder
ADHD: Attention-Deficit/Hyperactivity Disorder
ADNP: Activity- Dependent Neuroprotector Homeobox
ARID1B: AT-rich interaction domain 1B
ASD: Autism Spectrum Disorder
ATM: Ataxia-Telangiectasia Mutated
ATRX: Alpha-thalassemia mental retardation syndrome X-linked
BC: Breast Cancer
BD: Bipolar Disorder
CHD8: Chromodomain helicase DNA binding protein 8
CI: Confidence Interval
CRC: Colorectal Cancer
DNMT3A: DNA (cytosine-5-)-methyltransferase 3 Alpha
ERK: Extracellular Signal-Regulated Kinase
FOXP1: Forkhead Box P1
GAD: Generalized Anxiety Disorder
GWAS: Genome-Wide Association Study
HDG: Homogentisate 1,2 dioxygenase
HPA: Hypothalamic-Pituitary-Adrenal
HR: Hazard Ratio
ID: Intellectual Disability
MAPK: Mitogen-Activated Protein Kinase
MDD: Major Depressive Disorder
MR: Mendelian Randomization
mTOR: mechanistic Target of Rapamycin Kinase
NF1: Neurofibromin 1
NHI: National Health Insurance
NOTCH: Notch Receptor 1
OR: Odds Ratio
PAK: p21-Activated Kinase
PI3K: Phosphoinositide 3-Kinase
PTEN: Phosphatase and Tensin Homolog
SETD2: SET domain containing 2, Histone Lysine Methyltransferase
SIR: Standardized Incidence Ratio
SMARCA4: SWI/SNF-Related, Matrix-Associated, Actin-Dependent Regulator of Chromatin, Subfamily A, Member 4
SMARCB1: SWI/SNF-Related, Matrix-Associated, Actin-Dependent Regulator of Chromatin, Subfamily B, Member 1
SNP: Single Nucleotide Polymorphism
TCF7L2: Transcription Factor 7-Like 2
TGF-β: Transforming Growth Factor Beta
TSC1: Tuberous Sclerosis Complex 1
TSC2: Tuberous Sclerosis Complex 1
WNT: Wingless/Int-1
